# Community resilience indicators to inform geospatial health analyses: curating the resilience domain of the North Carolina multi-stressors database (NCMSD)

**DOI:** 10.3389/fpubh.2026.1821678

**Published:** 2026-06-03

**Authors:** Allison C. Spring, Sarah L. Miller, Cailee E. Harrington, Ellie Jiang, P. Grace Tee Lewis, Rebecca C. Fry, Lauren A. Eaves, Julia E. Rager

**Affiliations:** 1 Department of Environmental Sciences & Engineering, UNC Gillings School of Global Public Health, Chapel Hill, NC, United States; 2 Curriculum in Toxicology & Environmental Medicine, UNC School of Medicine, Chapel Hill, NC, United States; 3 Environmental Defense Fund, Austin, TX, United States; 4 Institute for Environmental Health Solutions, UNC Gillings School of Global Public Health, Chapel Hill, NC, United States

**Keywords:** built environment, data-driven research, geospatial, resilience, social determinants of health

## 1 Introduction

This study curated a geospatial database of tract-level resilience indicators to form the Resilience Domain of the North Carolina Multi-Stressors Database (NCMSD). NCMSD represents a larger effort currently being organized by our team, aimed to characterize multiple facets of vulnerability and resilience to factors affecting the health and wellbeing of people living in the state of North Carolina (NC). This larger database will be published and made publicly available after individual domains are finalized, such as the current Resilience Domain.

Here, we define resilience as the community resources and social structures that collectively strengthen and sustain community health and prevent, manage, or reduce the impact of adverse events. This definition represents a reframing of the classical view that resilience is the individual-level capacity to endure or recover from adversity (1). While resilience has been used in the past to task individual community members with becoming more “resilient” to public health challenges and natural disaster vulnerabilities (2), here, we apply the concept of resilience to describe broader social structures and community resources that could promote public health, as has been more recently proposed (3). We specifically focused on representative indicators across the following categories: (i) health and wellness, (ii) social and economic, (iii) infrastructure, and (iv) political capital, with indicators prioritized that addressed community resources and strengths relevant to human health. These categories are in accordance with recent community resilience frameworks that holistically capture community resources and capacity (1, 4, 5). While other efforts have sought to compile these indicators, they have been limited by the varying spatial and temporal scales at which data on resilience indicators are available and the patchwork of sources of these data, which include governmental agencies, non-profit organizations, and advocacy groups (6–13). Through the creation of this database, we seek to enable the integration of measures of community resilience into public health research, starting with NC.

To create this database, we established criteria for prioritizing resilience indicators, including data quality, non-missingness, geographic variation, interpretability, geospatial resolution, and lack of redundancy between indicators. When possible, indicators were prioritized that had low missingness (< 5%) and were available geospatially at the census tract level. Composite measures such as risk indices were generally not included due to limitations in interpretability. We performed basic statistical analyses, including correlations between measures, to identify resilience indicators that captured meaningful variation across the state without being redundant. Indicators with values outside the expected range, such as negative percentages, were excluded due to concerns about data quality and interpretability unless explicitly defined in the source codebook to represent missingness or another condition. This method may be applied in the future to create an updated database of resilience indicators or to other geographies as data becomes increasingly available. Collectively, this process generated a database that can be incorporated into future studies to inform public health burden and potential interventions.

## 2 Materials and methods

### 2.1 Searching for existing datasets

Table 1 lists all included indicators and their sources. Prior experience working with resilience datasets, expert feedback, and literature searches spanning peer-reviewed publications, books and gray literature (e.g., governmental reports, search engine queries) informed potential datasets for inclusion within the Resilience Domain of NCMSD. For subject-specific datasets, such as those mentioned in existing resilience frameworks, we used standard search engine searches to identify and obtain the datasets. We identified numerous existing sources of tabulated data to consider for inclusion within our resilience dataset, including the Agency for Toxic Substances and Disease Registry Environmental Justice Index (ATSDR EJI), Centers for Disease Control and Prevention and Agency for Toxic Substances and Disease Registry Social Vulnerability Index (CDC/ATSDR SVI), Centers for Disease Control and Prevention Population Level Analysis and Community EStimates (CDC PLACES) and National Environmental Public Health Tracking (CDC NEPHT), Department of Energy Low-income energy affordability (DOE LEAD) Tool, Environmental Protection Agency Environmental Justice Screening and Mapping Tool (EPA EJScreen), Federal Emergency Management Agency Community Resilience Challenges Index (FEMA CRCI), Healthy Communities NC, myFutureNC, ResilientNC, Robert Wood Johnson Foundation (RWJF), US Census Bureau American Community Survey (ACS) and the US Climate Vulnerability Index (US CVI) developed in partnership between the Environmental Defense Fund and researchers at Texas A&M University (11). Several indicator datasets were available through multiple sources. The source cited for each indicator in Table 1 reflects the source from which we retrieved the data and may not be the original source or curator of the dataset, which are noted in parentheses when identified. Datasets were downloaded between October 2024 and January 2025.

**Table 1:** Resilience indicators by category.

Health and wellness				
Indicator	Description	Timeframe	Processing (if applicable)	Source
Health Insurance	Inversion of lack of health insurance among adults aged 18–64 years as percentage	2019	Inverted data on current lack of health insurance	CDC PLACES
Hospital Beds	Number of hospital beds per 10,000 people	2016	Assigned county values to each census tract in county, assigned missing values as 0 based on assumption that there were no hospitals in these counties	US CVI [CDC NEPHTN]
Medical Practitioners	Number of medical practitioners per 1,000 people	2020	Converted 2020 to 2010 census tracts performing weighted averages by the population count	FEMA CRCI
Mental Health	Inversion of the percentage of individual reporting not good mental health	2015–2019	Inverted data to have percentage who did not report “not good” mental health	ATSDR EJI
Food Access	Inversion of the percentage of the population in the county who do not have access to food (food insecure)	2019	Assigned county values to each census tract in county, inverted data on food insecurity	US CVI [County Health Rankings & Roadmaps (24)]
Lack of Binge Drinking	Inversion of the model-based estimate of percentage of adults who have had five or more drinks (men) or four or more drinks (women) on an occasion in the past 30 days	2019–2021	Assigned county values to each census tract in county, inverted data on binge drinking	Healthy Communities NC [CDC PLACES]
Non-Smoking	Inversion of the model-based estimate of percentage of adults who have smoked ≥100 cigarettes in their lifetime, currently smoke every day or some days	2019–2021	Assigned county values to each census tract in county, inverted data on smoking	Healthy Communities NC [CDC PLACES]
Physical Activity	Inversion of the model-based estimate of percentage of adults who have not participated in any physical activities or exercises, other than a regular job, during the past month	2019–2021	Assigned county values to each census tract in county, inverted data on physical inactivity	Healthy Communities NC [CDC PLACES]
Adequate Sleep	Inversion of the model-based estimate of percentage of adults who get, on average, less than 7 hours of sleep during a 24-hour period	2019–2021	Assigned county values to each census tract in county, inverted data on inadequate sleep	Healthy Communities NC [CDC PLACES]
Non-Disabled	Inversion of the percentage of civilian noninstitutionalized population with a disability estimate	2015–2019	Replaced “−666666666” with NA, inverted data on disabled	ATSDR EJI
**Social and Economic**				
Indicator	Description	Timeframe	Processing (if applicable)	Source
Social and Civic Organizations	Number of social/civic organizations per 1,000 people	2003–2017	NA	US CVI [University of Michigan Institute for Social Research (25)]
Religious Institutions	Number of religious institutions per 1,000 people	2003–2017	NA	US CVI [University of Michigan Institute for Social Research (25)]
Library Access	Percent of census tract with a proximity to public library within a 5km centroid radius	2017	Assigned missing values to 0 based on assumption that census tract is not within radius and assigned 2 outliers above 1 to 1, assuming overlap between areas served by libraries	US CVI [Institute of Museum and Library Services (26)]
School Performance	Inversion of the percent of schools receiving a state-designated status of ‘low-performing'	2018–2019	Inverted	Healthy Communities NC [myFutureNC]
School Attendance	Inversion of the percentage of public school students who missed more than 10% of school days in a school year	2018	Inverted data on chronic school absenteeism and county values assigned to each census tract in county	Healthy Communities NC [myFutureNC]
Youth Engagement	Inversion of the percentage of population ages 16 to 19 not enrolled in school and not working	2020–2021	Inverted “Disconnected Youth”, averaged values from 2020–2023, and converted 2020 to 2010 census tracts by performing weighted averages by the population count aged 16–19 years old	Healthy Communities NC [ACS]
Employment	Inversion of the percent of population in the labor force that is unemployed	2015–2019	Inverted the percentage that was unemployed	ATSDR EJI
Economic Diversity	Inversion of the percentage of workforce employed in predominant sector	2020	Converted 2020 to 2010 census tracts performing weighted averages by the population count and inverted	FEMA CRCI
Income Equality	Inversion of the Gini Index of income inequality (income distribution across a population)	2020	Converted 2020 to 2010 census tracts performing weighted averages by the population count and inverted	FEMA CRCI
Gender Pay Equity	A ratio of women's median earnings to men's median earnings for all full-time, year-round workers, presented as cents on the dollar	2020–2021	County values assigned to each census tract in county	Healthy Communities NC [RWJF]
Residential Desegregation	Inversion of the index of dissimilarity where higher values indicate greater residential segregation between non-white and white county residents	2020–2022	Inverted metric of residential segregation and converted 2020 to 2010 census tracts by performing weighted averages by the population count	Healthy Communities NC [RWJF]
Housing Affordability	Percent of all housing units, including renters and owners, with monthly housing costs less than 30% of monthly income	2020-2022	Inverted percent of occupied housing units with monthly housing costs more than 30% of monthly income	Healthy Communities NC [RWJF]
**Infrastructure**				
Indicator	Description	Timeframe	Processing (if applicable)	Source
Non-Mobile Homes	Inversion of the percentage of all housing units that are mobile homes	2020	Inverted percentage of mobile homes, and converted 2020 to 2010 census tracts and performing weighted averages by the number of households	FEMA CRCI
Owner-Occupied Homes	Percentage of all housing units that are owner-occupied	2020	Converted 2020 to 2010 census tracts performing weighted averages by the number of households	FEMA CRCI
Housing Age	Inversion of the percentage of all housing units that were built before 1980	2015–2019	Inverted data on houses built after 1980	ATSDR EJI
Energy Affordability	Inversion of the average percent of median annual income that households pay for electricity and gas bills	2018–2022	Converted 2020 to 2010 census tracts performing weighted averages by the population and inverted	DOE LEAD Tool
Internet Access	Percentage of households with internet access	2013–2017	Percent of household with no internet access, inverted	Healthy Communities NC [ACS]
Computer Access	Percentage of households with computer access	2018–2022	Converted 2020 to 2010 census tracts performing weighted averages by the number of households	ResilientNC
Smart Phone Access	Inversion of the percentage of households without a smartphone	2020	Inverted percentage of households without a cell phone, and converted 2020 to 2010 census tracts and performing weighted averages by the number of households	FEMA CRCI
Greenspace	Proportion of census tract's area within 1-mi buffer of green space	2015-2019	NA	ATSDR EJI
Public Transit	Public transit performance score defined by how connected and accessible public transit is, and how often service comes; with lower scores indicating lower resilience	2019	NA	US CVI [All Transit (27)]
Walkability	A nationwide geographic data resource that ranks tract according to their relative walkability	2015–2019	NA	ATSDR EJI
Bikeability	Index of bikeability: described as how easy it is to bike to local destinations; with lower bikeability score indicating lower resilience	2022	NA	US CVI [Walkscore (28)]
Vehicles Access	Inversion of the percentage of households with no vehicle available	2020	Inverted percentage with no vehicle access and converted 2020 to 2010 census tracts performing weighted averages by the number of households	FEMA CRCI
**Political Capital**				
Indicator	Description	Timeframe	Processing (if applicable)	Source
Voter Turnout 2020	Population-adjusted voter turnout rate among voting eligible population in the 2020 election per 100,000 people	2020	NA	US CVI [MIT Election Lab (29)]

Indicators by resilience category, data source, and data processing steps performed for integration within the curated database. Data sources were accessed from October 2024 to January 2025. Within the Description and Processing columns, explanations are included on whether the indicator was inverted, to standardize each indicator such that a higher level was indicative of increased resilience. Data sources shown in brackets in the last column represent other identified sources of the same data. However, the listing of alternate sources is not exhaustive, and data retrieved from those sources may have different values compared to the selected data due to differences in data processing performed by different sources.

### 2.2 Organizing resilience indicator temporality and geospatial units

To align timeframes across different indicators, for each indicator, the most recent data release year that used the 2010 census tracts was prioritized when available. When data were available for multiple years that used the 2010 census tracts, values were averaged to minimize the missingness of the reporting for a single year. This led to the inclusion of data for a similar timeframe across indicators, as outlined in Table 1.

For indicators that were reported using the 2020 census tracts, 2020 Comparability Relationship File Record Layouts available through the US Census Bureau were used to determine values corresponding to the 2010 census based on weighted averages using the land area, the number of households, or population, depending on the metric (detail provided in Table 1) (9). For example, vehicle access was reported per household, and therefore, average vehicle access was calculated by weighting by the number of households in each 2020 census tract. Similarly, metrics reported with units of percent of the population were weighted by population in each 2020 census tract to calculate 2010 values. While indicators that were available at the census tract level were prioritized to reflect the granularity of community resilience, for indicators such as school performance and voter turnout, it was not possible to measure such characteristics within a census tract. Therefore, data available with county-level resolution was sourced, and the same value for a given indicator was applied to each census tract within a county. This approach does not account for within-county variation across such indicators and thus may not reflect true census tract conditions and potentially mask more localized disparities. For ease of interpretation, the directionality of all indicators was standardized, such that a higher level was indicative of increased resilience.

### 2.3 Data organization decisions and quality assurance amongst research team

The prioritization of indicators for the Resilience Domain of NCMSD was an iterative process that involved QA/QC from all co-authors. After potential indicators were sourced by AS and EJ, the relevance of indicators was independently reviewed by SM, who identified indicators that may not have a directionality associated with increased resilience or that were difficult to interpret for resilience directionality. For example, unpaid labor, a measure of the percentage of the population working as an unpaid family member, was removed from the considered indicators. While unpaid labor could be a source of support to individuals and societal infrastructure, it may also be disproportionately experienced by some community members and may reflect social inequities (14, 15). Therefore, it is challenging to interpret associations between resilience and unpaid labor. Various methods were also used to evaluate potential resilience indicators based on the available data. This process included performing basic descriptive statistics and graphing values by county and census tract to understand the range of values and potential outliers and assess data quality. Data quality concerns and relevant processing were discussed with all co-authors to form a consensus surrounding all methods employed in generating the Resilience Domain of NCMSD. Sourced indicators that were not ultimately incorporated within the Resilience Domain of NCMSD are summarized in Supplementary Table 1.

### 2.4 Data analysis script and workflow

**Figure 1:**
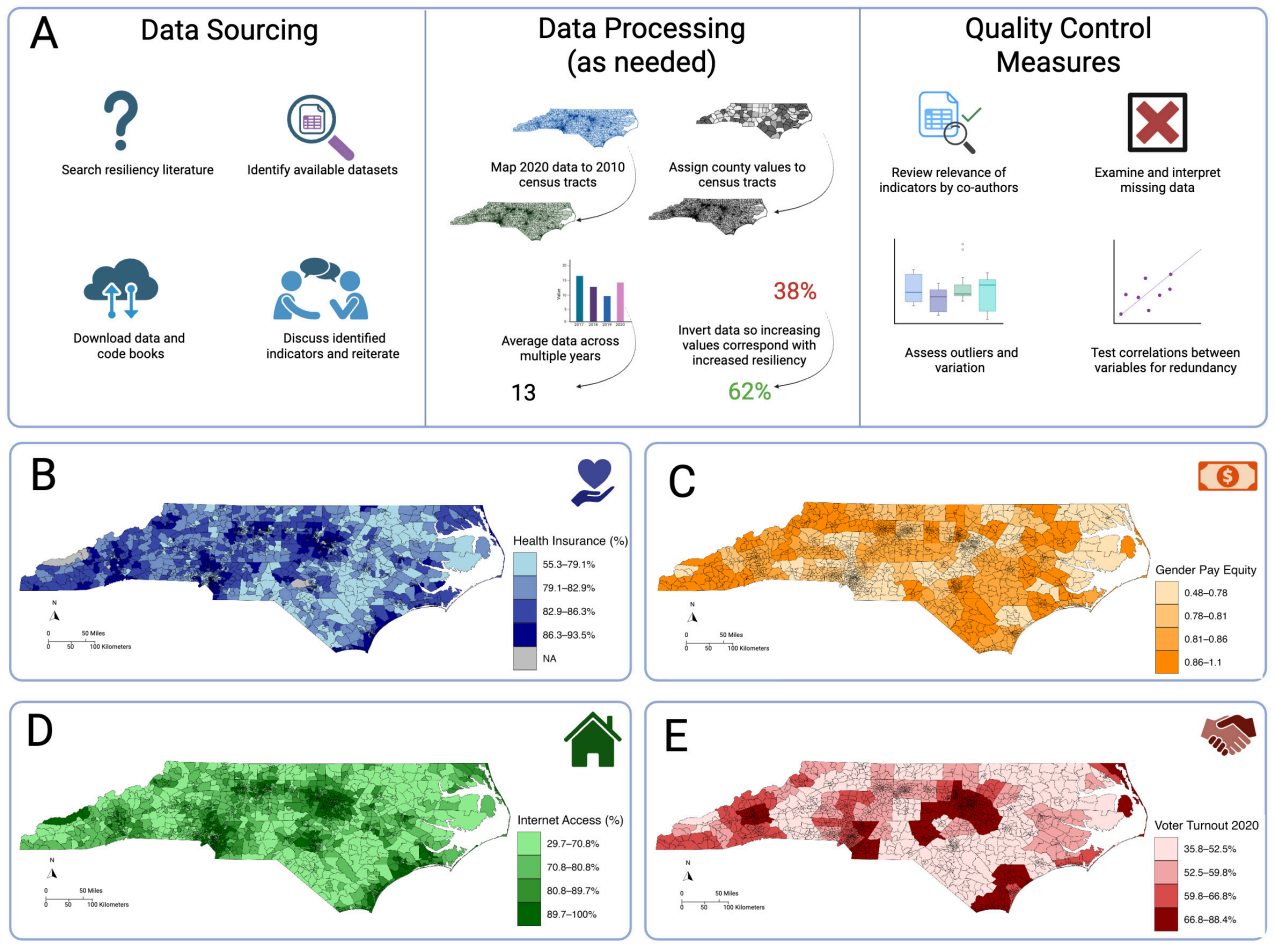
Curating the resilience domain of the North Carolina Multi-Stressors Database **(A)** Data sourcing, processing, and quality control measures. Aspects of the data organization process for indicators within the resilience domain of the NC Multi-Stressors Database (NC MSD) are summarized. Notably, different data processing was required for different indicators depending on the geospatial and temporal resolution and whether data was provided for 2010 or 2020 census tracts, as further detailed in this data report manuscript. **(B)** Health insurance data in the health and wellness category of the resilience domain. The average percentage of individuals, age 18-64, with health insurance per NC census tract, displayed by quartile. **(C)** Gender pay equity data in the social and economic category of the resilience domain. The ratio of women's median earnings to men's median earnings for all full-time, year-round workers in a county, displayed by quartile. **(D)** Internet Access data in the infrastructure category of the resilience domain. The percentage of households with internet access per NC census tract, displayed by quartile. **(E)** 2020 Voter turnout data in the political capital category of the resilience domain. Population-adjusted voter turnout rate among voting eligible population in the 2020 election by county, displayed by quartile.

All data manipulation and analysis were performed in R Studio using R version 4.4.2. General packages that were used throughout the analysis included *tidyverse* (16) and *dplyr* (17) in addition to baseline R packages. An overview of the data processing workflow is shown in Figure 1. Basic statistics were calculated for all selected resilience indicators, including the mean, standard deviation, median, minimum, maximum, interquartile range, 25th and 75th percentiles, and percent of missingness. Some datasets had undergone prior processing before integration into NCMSD. For example, missing values in the US CVI were imputed with the median of nationwide data for some indicators. Therefore, the percentage of census tracts that were imputed in NC was also analyzed. Pearson correlation coefficients were calculated between indicators based on pairwise complete observations using the *corrplot* package, ordering indicators based on the first principal component. All scripts are publicly available on the UNC-SRP GitHub site (18).

## 3 Data description and analysis

### 3.1 Health and wellness

Health and wellness indicators were extracted from the ACS, ATSDR EJI, CDC/ATSDR SVI, CDC PLACES, EPA EJ Screen, FEMA CRCI, Healthy Communities NC, and US CVI (6–13). These health and wellness measures are reflective of human capital and health vulnerabilities, which are vital to community resilience because they represent the baseline health of individuals who drive response and the capacity of community resources for recovery efforts (1). Selected indicators included health insurance (see Figure 1B), measured as the percent of adults aged 18–64 who have health insurance (2019) (8), the number of medical practitioners per 1,000 people in a census tract (2020) (6), and the number of hospital beds per 10,000 people in a county (2016) (11). Estimates of the percentage of the civilian noninstitutionalized population with a disability and the percentage of individuals reporting not good mental health were reported by EJI for 2015–2019 (12). Access to food was represented as the inverse of percent of people within a county who were food insecure in 2019 (11). Individual health behaviors that affect wellness, including sleep, physical activity, smoking, and binge drinking, were reported by Healthy Communities NC from PLACES data for 2019–2021 (13). These indicators were defined as the percentage of adults who get on average at least 7 h of sleep per night, who have participated in physical activities in the past month, who have not smoked more than 100 cigarettes in their lifetime, and who have had fewer than five drinks if male or four drinks in female on an occasion in the past 30 days, respectively (8).

### 3.2 Social and economic

Social and economic indicators were extracted from the ACS, FEMA CRCI, Healthy Communities NC, myFutureNC, and RWJF (6, 9, 13, 19–21). This domain encompasses the networks of local institutions and interpersonal relationships, along with the financial resources and economic opportunities that together support community wellbeing and development (1). These indicators included the following: school performance, school attendance, and youth engagement (13), social and civic organizations (11), and religious institutions (11), living near a public library (11), economic stability (6, 13), and community equality (6, 13, 21). School performance was assessed as the percentage of schools not receiving a state-designated status of low-performing (2018–2019) (13). School attendance, defined here as the lack of chronic absenteeism, is the percentage of public-school students who missed < 10% of school days in a school year (2018) (13), and youth engagement is the percentage of the population aged 16 to 19 enrolled in school or working (2020–2021) (13). Social and civic organizations and religious organizations were defined as the number of organizations per 1,000 people from 2003 to 2017 (7, 11). Library access, defined as living near a public library, was included as the percentage of a census tract that lives within three miles of a public library (2017) (7, 11). This serves as a measure of community space and access to resources. Economic stability metrics included the percent of people who are employed (2015–2019) (12) and the percent of the workforce employed not in the dominant sector (2020) (6). Housing affordability for renters and homeowners was defined as the percentage of households with housing costs less than 30% of their income (2020–2022) (13). Lastly, measures of community equality included the Gini Index of income inequality, a measure of income distribution across the population (2020) (6), gender pay equity (see Figure 1C) (13), a ratio of women's median earnings to men's median earnings for all full-time, year-round workers (2020–2021) (13, 21), and the residential segregation index of dissimilarity (2020–2022) (13, 21), a measure of the residential segregation between non-White and White county residents (21).

### 3.3 Infrastructure

Infrastructure indicators evaluated critical aspects related to the adequacy of housing (6, 13); transportation (7, 13); energy (22); access to greenspace (12); and access to the internet (13), smartphones (6), and computers (23). These indicators were sourced from ATSDR EJI, the DOE LEAD Tool, FEMA CRCI, ResilientNC, and US CVI (6, 11, 12, 22). These indicators were included to represent the accessibility of community resources and connectivity, from physically navigating space to having means of communication and access to information. Housing indicators included the percentage of all housing units that were owner-occupied (2020) (6), the percentage of renter and owner-occupied housing units that were not mobile homes (2020) (6), and the percentage of renter and owner-occupied housing units that were built after 1980 (2015–2019) (12). Transportation was measured as the percentage of households without a vehicle (2020) (6), relative walkability (2015–2019) (12), bikeability scores (2022) (11), and public transit performance scores (2019) (11). The affordability of energy was captured as the average percent of median annual income that households paid for electricity and gas bills (2018–2022) (22). As a proxy for access to parks and green space, we selected the proportion of tract area within a 1-mile buffer of green space (2015–2019) (12). Lastly, smartphone, computer, and internet access were measured as the percentage of households with a smartphone (2020) (6), the estimated percent of households with one or more members of the household owning or using a computer (2018–2022) (23), and the average percent of households with internet access (2013–2017) (11), respectively (see Figure 1D).

### 3.4 Political capital

In the final resilience category, the rate of voter turnout in the 2020 presidential election among those eligible to vote (11) was used as an indicator of political capital reflective of enfranchisement and civic engagement (see Figure 1E) (1). This indicator was selected based on the resilience activation framework proposed by Abramson et al. (1). Other metrics, which were considered for this domain, included access to people in leadership or the effectiveness and equity of government infrastructure; however, relevant data for such indicators was not identified at a census or country level tract for NC.

### 3.5 Resilience indicator distributions and correlations

Basic summary statistics are summarized for each indicator in Supplementary Table 2. Correlations between indicators by domain are presented in Supplementary Figure 1, ordered by the first principal component value. Notably, this ordering did not group indicators by resilience category, indicating that there were correlations both within and across resilience categories. For example, physical activity was strongly positively correlated with non-smoking (*r* = 0.94), health insurance (*r* = 0.90), internet access (*r* = 0.84), mental health (*r* = 0.84), adequate sleep (*r* = 0.81), and energy affordability (*r* = 0.80). Moreover, housing affordability, classified as a social and economic indicator, and owner-occupied homes, an infrastructure indicator, were positively correlated (*r* = 0.72). Other related indicators, such as computer access and smartphone access, were also positively correlated (*r* = 0.79), as expected. Several of these indicators were sourced from different data sources, further validating the approach used to identify, process, and combine indicators. In general, resilience indicators were positively correlated with other resilience indicators, except for library access, religious organization, and lack of binge drinking. For instance, there were negative correlations between the lack of binge drinking and physical activity (*r* = −0.74) and internet access (*r* = −0.68).

## 4 Concluding remarks

This dataset represents an important compilation of indicators that can now be used to inform resilience across the state of NC, now poised for integration into NCMSD. While this iteration of the dataset is novel and informative, it is not without limitations. First, while we prioritized data that was available at the census tract geospatial resolutions, for several indicators, due to data availability, the same value was assigned to each census tract in a county; these values may not accurately reflect exposures within a census tract. Moreover, while the resulting database captures the geospatial variation in community resilience indicators, it does not capture temporal variation. Furthermore, the majority of values came from documented measures from the 2010 census tracts, while pulling data from more recent years when needed to assist in data completeness. As data becomes increasingly updated and available from more recent years, we hope to continue to update this resource to continue to inform resilience indicators across the state. In presenting this method of identifying and organizing relevant sources of resilience data, this effort can now be repeated in the future to reflect changes in these data and downstream interpretations that could assist in public health studies and decision-making. Future efforts will also combine additional indicators to capture community resilience as more data become available in the coming years.

## Data Availability

The datasets presented in this study can be found in online repositories. The names of the repository/repositories and accession number(s) can be found below: UNC-SRP Dataverse at: https://dataverse.unc.edu/dataverse/UNCSRP.
