# Supplementary material for: Community resilience indicators to inform geospatial health analyses: curating the resilience domain of the North Carolina multi-stressors database (NCMSD)
Source: Front Public Health. 2026 Jun 3;14:1821678. doi: 10.3389/fpubh.2026.1821678 (PMC13274430; doi:10.3389/fpubh.2026.1821678)
Supplement: Supplementary file 1 [file Supplementary_file_1.docx]

Supplementary Material


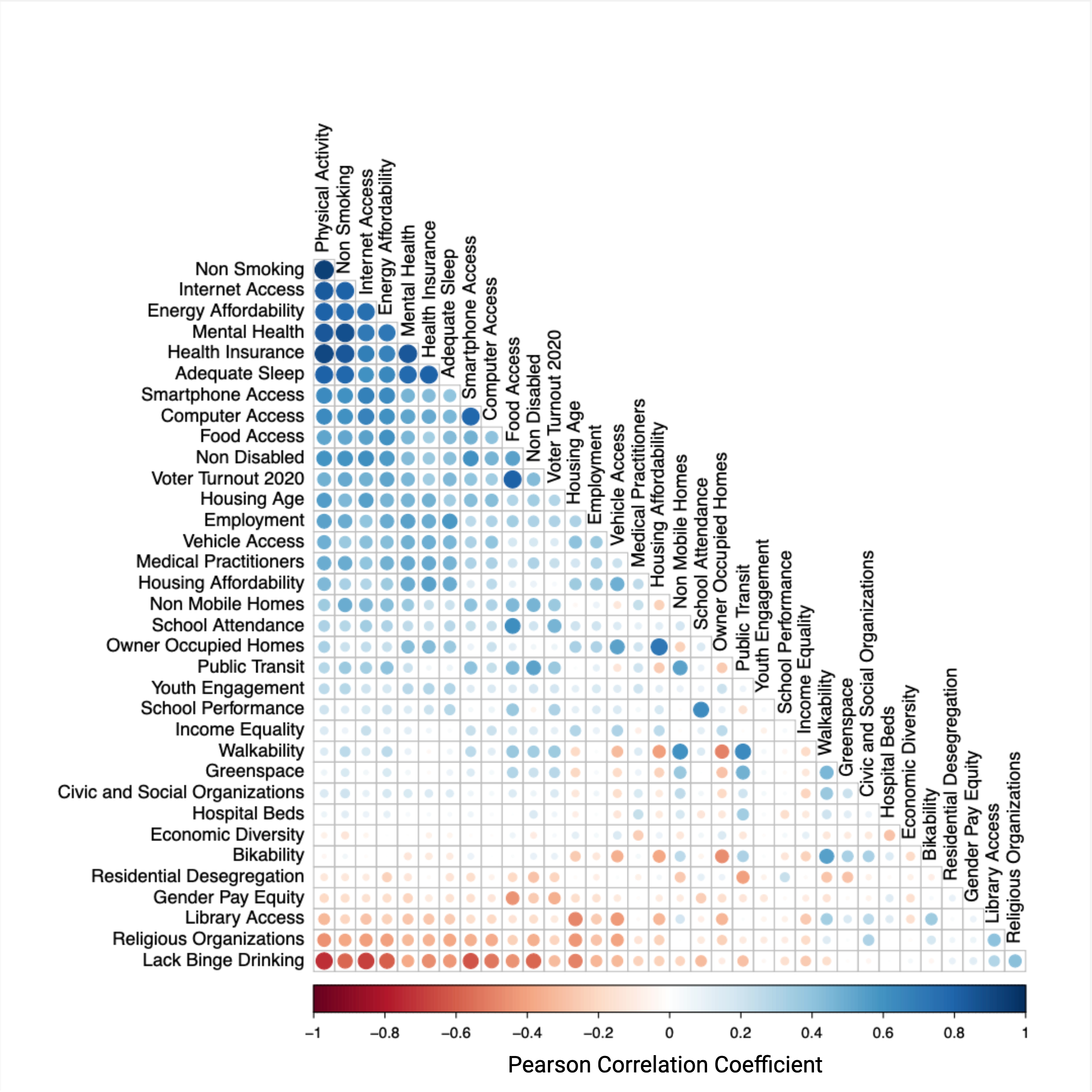


**Supplementary Figure 1. Correlations between resiliency indicators**. Pearson correlations were calculated for pairwise complete observations using census tract values for resiliency indicators. Variables are ordered using the first principal component.
